# TSPO: a conserved mitochondrial conduit linking cellular homeostasis to disease

**DOI:** 10.1038/s41419-026-09126-w

**Published:** 2026-08-18

**Authors:** Asif Rasheed, Eva Sidlauskaite, Michelangelo Campanella

**Affiliations:** 1 Department of Biomedical Sciences, University of Padua, Padua, Italy; 2 William Harvey Research Institute, Queen Mary University of London, London, United Kingdom; 3 Institute Gustave Roussy, UMR 9018-CNRS, Villejuif, France

**Keywords:** Mitochondria, Mechanism of action, Parkinson's disease, Targeted therapies, Endocrine system and metabolic diseases

## Abstract

The translocator protein (TSPO) is an evolutionarily conserved 18 kDa protein localised to the outer mitochondrial membrane and increasingly recognised as a multifunctional regulator of cellular homeostasis. Although initially identified as the peripheral benzodiazepine receptor, TSPO has since been implicated in a range of mitochondrial processes. This review integrates current evidence on TSPO function across evolution and in mammalian systems, with a focus on its interactions with mitochondrial gatekeeping proteins. TSPO has been implicated in regulating cholesterol transport, redox homeostasis, calcium (Ca^2+^) signalling, and mitochondrial quality control. Mechanistically, TSPO has been proposed to modulate Ca^2+^ flux and reactive oxygen species (ROS) production through VDAC1-associated pathways, linking mitochondrial dynamics to cellular stress responses. We also evaluate the context-dependent effects of TSPO ligands across disease models. Collectively, these findings position TSPO as a potential integrator of mitochondrial signalling with important implications for understanding disease mechanisms and therapeutic targeting.

## Facts

- What is the primary physiological function of TSPO: cholesterol transport, cholesterol sensing, or enzymatic regulation?
- How does TSPO mechanistically regulate mitochondrial Ca^2+^ homeostasis and redox signalling pathways?
- What molecular and cellular determinants govern the context-dependent effects of TSPO on autophagy and mitophagy?
- Does TSPO play a causal role in disease pathogenesis, or does its upregulation primarily reflect cellular stress and inflammatory responses?
- Can TSPO-associated signalling pathways be harnessed for precision medicine despite genetic polymorphisms and ligand-specific variability?

## Introduction

The 18-kDa translocator protein (TSPO) is a transmembrane protein localised to the outer mitochondrial membrane (OMM). It is ubiquitously expressed across tissues, with particularly high levels in the brain, adrenal glands, and heart [1, 2]. Since its identification in 1977, the understanding of TSPO biochemistry has evolved considerably. It has been classified as a receptor, a transporter, a component of multiprotein complexes, and more recently as a putative enzyme.

Despite these discrepancies, TSPO has remained a key mitochondrial target for pharmacological investigation, owing to the extensive development of compounds designed to trace and modulate its activity.

TSPO was originally termed the Peripheral Benzodiazepine Receptor (PBR) following the discovery of its ability to bind benzodiazepines in peripheral tissues [1]. The denomination was later abandoned when it became evident that TSPO does not function as a classical receptor. Instead, it may indirectly contribute to GABAergic signalling, particularly via chloride influx, through mitochondrial production of pregnenolone and allopregnanolone. This realisation prompted a reassessment of TSPO’s proposed role in neurotransmission and its putative receptor-like function [3].

The currently adopted nomenclature, TSPO, reflects its capacity to act as a high-affinity cholesterol-binding protein via a conserved cholesterol recognition/interaction amino acid consensus (CRAC) domain located in its C-terminal region [4]. Beyond cholesterol binding, TSPO cooperates with the steroidogenic acute regulatory protein (STARD1) to facilitate cholesterol accumulation at the inner mitochondrial membrane, thereby justifying the designation “translocator protein”.

TSPO has been proposed to constitute a critical upstream element in steroid and neurosteroid biosynthesis [5], a hypothesis that garnered substantial attention [6]. However, accumulating in vivo evidence has challenged the indispensability of TSPO in steroidogenesis, and this role remains debated, shifting attention toward alternative and still incompletely defined mitochondrial and cellular functions.

In addition to cholesterol, TSPO binds endogenous ligands such as protoporphyrin IX (PPIX) and participates in multiprotein complexes with mitochondrial and non-mitochondrial partners, including VDAC and ACBD3. Through these interactions, TSPO influences quality control mechanisms such as autophagy and mitophagy, as well as homeostatic cellular signalling pathways, including calcium dynamics. Consequently, TSPO is increasingly regarded as a multifunctional protein whose role extends beyond steroid synthesis to encompass adaptive cellular responses, including inflammation and pro-survival signalling [7].

More recently, TSPO has also been proposed to exhibit enzymatic activity as a cholesterol-sensitive oxygenase. This model represents a conceptual shift from the classical view of TSPO as a cholesterol transporter. However, it is highly recent and remains controversial and requires further validation in mammalian systems [8].

In pathological contexts, TSPO has attracted sustained interest due to its consistent upregulation in both acute and chronic conditions. Altered TSPO expression has been reported across a wide spectrum of diseases, including cancer [9], metabolic disorders [10], cardiovascular diseases [11], neurodegeneration [12], and neuropsychiatric conditions [13]. These observations suggest a contributory role for TSPO in disease onset and progression, underscoring the broad attention this mitochondrial protein has received over the years.

More recently, the TSPO acronym has been reinterpreted as *Tryptophan Sensory Protein* to reflect a proposed enzymatic function as an oxygenase. TSPO has recently been proposed to act as a cholesterol-activated enzyme, with cholesterol serving as both a ligand and a molecular sensor; however, this model remains highly preliminary and requires independent validation in mammalian systems [8]. Although still preliminary, this interpretation aligns with longstanding evidence implicating TSPO in redox homeostasis [14], including effects on both intra- and extramitochondrial sources of reactive oxygen species (ROS) and antioxidant pathways [15]. The dual involvement of TSPO in lipid handling and redox regulation has positioned it as a compelling target for molecular manipulation of cellular physiology and pathology. This opportunity has been extensively explored through the development of TSPO ligands. Such ligands are currently employed in experimental, preclinical, and clinical settings [16].

Beyond its translational relevance, TSPO biology is of fundamental interest due to its high degree of evolutionary conservation. TSPO homologues are found in prokaryotes and lower eukaryotes, offering valuable systems through which their core biochemical functions may be more fully elucidated. This manuscript begins by highlighting an evolutionary perspective, then addresses the cellular and molecular physiology of TSPO, and subsequently examines its roles in pathology.

Overall, the review aims to provide a balanced and critical overview of current knowledge on TSPO, to stimulate renewed interest and encourage novel interpretative frameworks for understanding its biological functions.

## Evolutionary conservation and structural organisation of TSPO

Most of the literature on the translocator protein (TSPO) has been derived from studies in mammalian systems, yielding substantial insights into its physiological and pathological roles. However, the evolutionary history of TSPO is far more ancient, originating in prokaryotes, where fundamental aspects of its structure, function, and biological significance can be discerned.

The TSPO coding sequence is remarkably conserved across evolution, from bacteria to mammals, encoding a small hydrophobic protein composed of five transmembrane helices enriched in conserved tryptophan residues [17]. Functional TSPO homologues have been identified throughout the phylogenetic tree. TSPO cDNA has been cloned from numerous species [18]. Although sequence conservation varies considerably across taxa, mammalian TSPOs share ~80% sequence identity, whereas similarity between mammalian and bacterial TSPOs is limited to 30–35%. In contrast, TSPOs from plants and cyanobacteria display lower and more variable homology (12–36%), with higher conservation observed among plant species themselves (38–85%) [19].

The erythroid-specific isoform TSPO2, present in mammals and birds, shares only ~35% sequence identity with canonical TSPO1, and additional TSPO-like isoforms have been identified in cyanobacteria and plants [20]. Notably, rat TSPO can functionally substitute for its bacterial counterpart in *Rhodobacter sphaeroides*, despite sharing only ~30% amino acid identity, indicating strong evolutionary conservation of TSPO function [21]. In humans, TSPO is encoded by a single-copy gene located on chromosome 22q13.31 and produces a 169-amino-acid protein that shares 81% sequence identity with murine TSPO [22].

Investigations in bacterial and lower eukaryotic models have provided critical structural and mechanistic insights that are often obscured in complex mammalian systems, thereby offering a foundational framework for understanding TSPO organisation, ligand-binding properties, and functional versatility in higher organisms.

A homologue of mammalian TSPO was first identified in the gram-negative photosynthetic bacterium *Rhodobacter*, considered an ancestral relative of mitochondria [23]. In *Rhodobacter*, TSPO regulates the metabolic switch from respiration to photosynthesis in response to oxygen tension and light availability, functioning as part of a sensory regulatory system that includes porphyrin translocation [24]. High-resolution structural insights into TSPO have been obtained from bacterial homologues. These include crystal structures of wild-type R. sphaeroides TSPO (RsTSPO), Bacillus cereus TSPO (BcTSPO), and mouse TSPO [25]. Despite limited sequence similarity, the TSPO three-dimensional structure appears more conserved than its primary sequence, as both *R. sphaeroides* and *B. cereus* TSPO adopt a similar five-helix architecture, supporting the existence of an evolutionarily conserved structural core [26].

Although no structures have yet been reported for plant or cyanobacterial TSPOs, conservation of key residues, particularly tryptophans and histidines, suggests preservation of the core five-helix fold [27]. Some plant and cyanobacterial TSPOs possess extended N-terminal regions that may form additional helices and confer species-specific functions [28]. TSPO oligomerisation further adds complexity: bacterial TSPOs exist as monomers or dimers depending on environmental conditions, and multiple oligomeric states have been observed within the same organism or tissue [19]. In *R. sphaeroides*, TSPO forms parallel dimers under specific growth conditions, as supported by crystallographic and cryo-EM analyses. Collectively, these observations suggest that TSPO oligomerisation reflects distinct functional states rather than a universally conserved structural property.

## TSPO interactions in mitochondrial gatekeeping

Mitochondrial gatekeeping molecules regulate the selective exchange of ions, metabolites, and signalling factors between mitochondria and the surrounding cellular environment, thereby preserving mitochondrial and cellular homeostasis [29, 30]. Key gatekeepers include proteins of the outer and inner mitochondrial membranes, such as the voltage-dependent anion channel (VDAC) and the translocase complexes of the outer and inner membranes (TOM and TIM). Through dynamic regulation of calcium (Ca^2+^) flux, redox signalling, metabolite transport, and the release of apoptotic factors, these systems critically determine mitochondrial bioenergetic efficiency, stress responsiveness, and cell fate. TSPO has been proposed to associate with several of these gatekeeping components and, under specific conditions, to exert regulatory control over their activity. A major focus has been the interaction between TSPO and the voltage-dependent anion channel (VDAC), which exists in three isoforms, with VDAC1 being the predominant and most extensively studied form [31]. Extensive evidence indicates that TSPO-VDAC1 interactions influence ion fluxes, reactive oxygen species (ROS) signalling, and mitochondrial quality control mechanisms [32]. In particular, TSPO has been shown to suppress mitophagy downstream of the PINK1–PARK2 pathway by inhibiting the ubiquitination of key substrates required for mitochondrial clearance [32] (see Autophagy section). Through regulation of mitochondrial ROS production, TSPO-VDAC1 interactions have been proposed to link mitochondrial signalling to mitochondria-dependent apoptosis [33, 34]. Excessive or unbuffered ROS can activate VDAC1, promoting VDAC1-mediated cytochrome c release from mitochondria [35]. The close physical proximity between TSPO and VDAC1 provides a structural basis for TSPO-derived ROS to modulate VDAC1 activity directly. Moreover, clustering or polymerisation of TSPO around VDAC1 may generate localised ROS microdomains near the channel, thereby amplifying apoptotic signalling [36].

In parallel, elevations in cytosolic Ca^2+^ can induce intracellular acidification [37] and promote VDAC1 oligomerisation [38], further facilitating programmed cell death. VDAC serves as the principal Ca^2+^ conduit of the outer mitochondrial membrane [39]. Therefore, TSPO-VDAC1 interactions may influence Ca^2+^ homeostasis and downstream signalling pathways, as mechanistically detailed in the redox and Ca^2+^ signalling section. Consistent with the notion, TSPO ligands have been shown to modulate both mitochondrial and cytosolic Ca^2+^ dynamics [40].

Additional regulatory complexity arises from TSPO interaction with acyl-CoA binding domain-containing protein 3 (ACBD3) [41], a scaffold for cAMP-dependent protein kinase A (PKA). Through this interaction, TSPO may regulate VDAC activity via phosphorylation-dependent mechanisms (see Section ‘TSPO in Redox and Calcium (Ca^2+^) Signalling’), providing an additional TSPO-centred regulatory axis.

TSPO has also been implicated in regulating the opening of the mitochondrial permeability transition pore (mPTP). The mPTP is a high-conductance channel activated by Ca^2+^ overload and other stressors that induces mitochondrial swelling, membrane depolarisation, and cytochrome c release - although this association remains controversial. Early models proposed that the mPTP consisted of VDAC1 in the outer mitochondrial membrane, adenine nucleotide translocase (ANT) in the inner mitochondrial membrane, and cyclophilin D (CyD) in the matrix [42]. However, genetic ablation of ANT isoforms [43] or VDAC [44] failed to abolish mPTP formation, prompting the development of alternative models. Among these, the model proposing F₁F₀-ATP synthase dimerisation as the core pore-forming unit has gained major consensus [45].

Nevertheless, a contributory role for TSPO in facilitating mitochondrial membrane potential (ΔΨm) dissipation remains plausible, analogous to other VDAC1-interacting proteins such as hexokinase (HK), DIDS, and G3139, which act as mPTP opening potentiators [46]. Evidence supporting TSPO involvement includes observations that anti-TSPO antibodies delay mPTP opening and inhibit the release of apoptosis-inducing factor (AIF) from isolated mitochondria. In addition, both exogenous TSPO ligands (e.g., Ro5-4864, PK11195) and the endogenous ligand protoporphyrin IX (PPIX) have been reported to modulate mPTP activity [47].

However, these ligand effects may reflect off-target actions, including interactions with mitochondrial F₁F₀-ATP synthase, and the classification of TSPO as a bona fide mPTP component remains controversial [48]. The translocase of the outer membrane (TOM) complex mediates the import of most nuclear-encoded mitochondrial proteins and comprises receptor subunits Tom20, Tom22, Tom70, and the β-barrel channel Tom40. Beyond protein translocation, TOM also facilitates lateral membrane insertion of select substrates. Given that TSPO is an α-helical outer mitochondrial membrane protein, TOM has been proposed to mediate TSPO targeting, insertion, or assembly within the membrane [49].

Nevertheless, studies examining TOM dependence in TSPO biogenesis have yielded conflicting results. Biochemical reconstitution and Blue-Native PAGE analyses identified TSPO within ~66 kDa assemblies consistent with interactions with import or assembly factors. However, RNA interference and functional assays showed that depletion of components of the translocase of the outer membrane (TOM) complex, including Tom20, Tom22, and Tom40, does not uniformly disrupt TSPO targeting or membrane insertion. These discrepancies suggest that TSPO may utilise alternative insertase or chaperone pathways, or that TOM dependence varies across species, experimental systems, or stages of biogenesis examined. Recent work has further suggested a functional coupling between TSPO and Tom40 that extends beyond protein import. Genetic and pharmacological studies in microglia and neuropsychiatric disease models indicate that Tom40 is required for specific downstream effects of TSPO ligands, including modulation of mitochondrial function, ROS signalling, and behaviour. These findings support a model in which TSPO functionally interfaces with the TOM machinery, even if Tom40 is not the exclusive mediator of TSPO membrane insertion [50]. Collectively, these interactions position TSPO as a multifunctional modulator of mitochondrial gatekeeping, integrating metabolic, redox, and signalling cues to fine-tune mitochondrial adaptability under physiological and pathological conditions.

## TSPO from cholesterol transport to steroidogenesis and beyond

Originally characterised for its role in mitochondrial cholesterol transport and steroid hormone biosynthesis, the translocator protein (TSPO) has long been regarded as a key regulator of steroidogenesis [51]. Over the past decades, however, accumulating evidence has substantially expanded this classical view, revealing TSPO as a multifunctional protein involved in a broad spectrum of cellular processes that extend well beyond steroid hormone production.

Consistent with its cholesterol-binding capacity (see Introduction), TSPO has been proposed to facilitate cholesterol transport into mitochondria, where pregnenolone synthesis occurs [52]. Structural and functional studies highlighted the importance of the CRAC domain, particularly residue Y152 in cholesterol binding, supporting a role for the TSPO C-terminus in mitochondrial cholesterol handling [53].

Cholesterol is the essential precursor for steroid hormones and bile acids. Therefore, tight regulation of its intracellular trafficking is critical for cellular and systemic homeostasis [52]. Cholesterol translocation to mitochondria represents a rate-limiting step in steroid biosynthesis, thereby influencing metabolic and immune functions [2, 54]. In this context, TSPO was proposed to function as part of a multiprotein complex involving the voltage-dependent anion channel (VDAC) and the adenine nucleotide translocator (ANT), facilitating cholesterol transport from the outer mitochondrial membrane (OMM) to the inner mitochondrial membrane (IMM).

TSPO was further proposed to interact with the steroidogenic acute regulatory protein (StAR; STARD1). Together, these proteins may form a functional complex that promotes cholesterol delivery to the inner mitochondrial membrane, where steroid synthesis is initiated [55]. Supporting the proposed model, expression of an OMM-anchored Tom20-StAR fusion protein enhances steroidogenesis, suggesting that StAR mitochondrial import is not required and that its activity is exerted at the OMM.

In these systems, TSPO knockdown markedly reduced steroidogenic output, leading to the conclusion that TSPO and StAR cooperate in mitochondrial cholesterol transfer [56]. The evidence gave rise to the influential model in which StAR functions as the “initiator” of cholesterol mobilisation, while TSPO acts as a “gate” controlling cholesterol entry into mitochondria.

By regulating mitochondrial cholesterol availability, TSPO was implicated in the biosynthesis of glucocorticoids, mineralocorticoids, and sex hormones [57]. Altered TSPO expression or function has been linked to endocrine disorders. Early genetic and pharmacological studies suggested that TSPO deficiency impairs steroidogenesis, whereas TSPO overexpression enhances steroid hormone production [58]. However, subsequent in vivo studies have challenged the indispensability of TSPO for steroid biosynthesis, indicating that its role may be modulatory rather than strictly essential and context-dependent.

Consistent with its involvement in cholesterol handling, defects in TSPO or StAR have been implicated in congenital lipoid adrenal hyperplasia, a rare disorder characterised by adrenal insufficiency and impaired steroid hormone synthesis [51]. Altered TSPO expression has also been reported in adrenal tumours and other malignancies associated with endocrine dysregulation [59], underscoring its clinical relevance.

Historically, the link between TSPO and steroidogenesis originated from observations that TSPO-binding compounds stimulated steroid production in Y1 adrenal tumour cells, later confirmed in MA-10 Leydig cells [60]. The endogenous TSPO-interacting protein acyl-CoA-binding protein (ACBP/ACBD1), formerly known as the diazepam-binding inhibitor, similarly enhanced steroid synthesis [61]. TSPO knockdown studies further supported a requirement for TSPO in steroid production, and identification of the CRAC motif provided a molecular basis for cholesterol binding [4, 62].

Nevertheless, key observations complicated this model. StAR-mediated cholesterol transport does not require mitochondrial import of StAR itself, as deletion of its N-terminal targeting sequence does not impair steroidogenesis [63]. Moreover, while OMM-tethered StAR enhances steroid synthesis, TSPO knockdown abolishes the effect, reinforcing a cooperative but OMM-restricted mechanism [56].

More recently, structural and biochemical evidence has fundamentally challenged the view of TSPO as a primary mitochondrial cholesterol transporter. Using detergent-free native membrane nanoparticles, Qiu and colleagues demonstrated that human TSPO1 functions as a cholesterol-dependent enzyme catalysing the oxidative cleavage of protoporphyrin IX into bilindigin [8]. In this model, cholesterol stabilises TSPO conformation and enzymatic activity rather than serving as a transported substrate. Notably, bacterial TSPO homologues exhibit cholesterol-independent activity, suggesting that cholesterol dependence evolved in eukaryotic TSPO. These findings suggest that TSPO may function as an enzyme regulating porphyrin metabolism and redox signalling, rather than as a dedicated mediator of mitochondrial cholesterol import, thereby challenging steroidogenesis-centred paradigms.

**Fig. 1:**
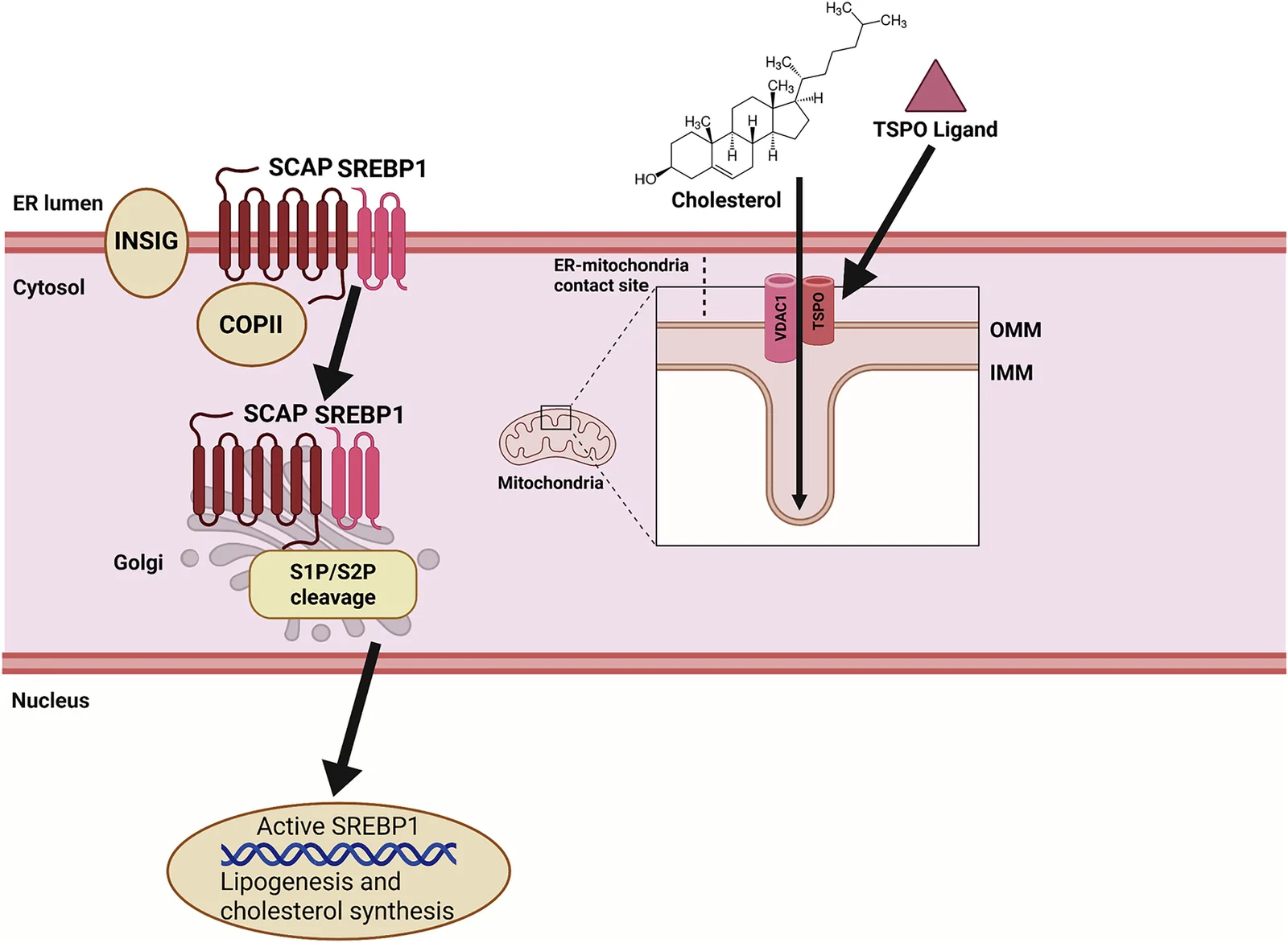
TSPO in cholesterol binding, translocation, and steroidogenesis. TSPO, located in the outer mitochondrial membrane, binds cholesterol and facilitates its translocation from the intermembrane space to the inner mitochondrial membrane, where cholesterol is converted to pregnenolone, the rate-limiting step in steroidogenesis. This links the TSPO function to endocrine and metabolic regulation. TSPO can also interact with exogenous ligands, which induce conformational changes that modulate mitochondrial and cellular processes. Beyond its role in mitochondrial function, TSPO-mediated cholesterol binding may have broader effects, such as reducing lipid accumulation at ER-mitochondria contact sites. The following reduction could promote dissociation of the SCAP-SREBP1 complex from the ER retention factor INSIG, enabling COPII-mediated trafficking of SREBP1 to the Golgi, where sequential cleavage by site-1 and site-2 proteases (S1P/S2P) releases the active N-terminal fragment. This fragment then translocates to the nucleus, where it induces the expression of genes involved in lipogenesis and cholesterol biosynthesis. Thus, TSPO may influence both mitochondrial and extra-mitochondrial cholesterol dynamics, indirectly facilitating SREBP1 activation. Created with BioRender.com.

The long-standing view of TSPO as an essential mediator of mitochondrial cholesterol transport and steroidogenesis has been increasingly challenged. Early pharmacological and knockdown studies supported a functional interaction between TSPO and the steroidogenic acute regulatory protein (STARD1), suggesting a cooperative role in promoting cholesterol delivery to the inner mitochondrial membrane [51, 52]. However, more recent genetic models have demonstrated that TSPO deletion does not universally impair steroid hormone synthesis in vivo, questioning its indispensability in this process [58, 64]. These findings suggest that TSPO may function as an enzyme regulating porphyrin metabolism; however, this model remains preliminary and requires independent validation in mammalian systems. In parallel, emerging structural and biochemical evidence has proposed alternative functions for TSPO, including a potential enzymatic role in porphyrin metabolism and redox regulation [8]. In addition, we speculate that increased TSPO expression, resulting in enhanced cholesterol binding, may influence sterol regulatory element-binding protein 1 (SREBP1) signalling by modulating lipid microdomains at endoplasmic reticulum-mitochondria contact sites. However, this hypothesis remains to be experimentally validated. Such a mechanism could potentially promote compensatory upregulation of cholesterol biosynthesis pathways under conditions of altered TSPO abundance (Fig. 1); however, this remains to be experimentally validated.

Beyond steroidogenic tissues, TSPO has been linked to cardiovascular disease, particularly atherosclerosis, in which cholesterol is a bona fide pathogenic factor. Atherosclerosis is characterised by chronic inflammation and cholesterol accumulation within the arterial walls. TSPO regulates cholesterol efflux from macrophages, limiting foam-cell formation and plaque progression [65]. Pharmacological targeting of TSPO and genetic TSPO deficiency in animal models attenuate atherosclerotic lesion development, highlighting TSPO ligands as potential anti-inflammatory and lipid-modulating therapeutic agents [15].

Similarly, the liver, a central organ of cholesterol metabolism, is influenced by TSPO activity. Altered TSPO expression has been reported in non-alcoholic fatty liver disease (NAFLD) and non-alcoholic steatohepatitis (NASH), implicating TSPO in disease pathogenesis [66]. Disrupted cholesterol homeostasis exacerbates hepatic steatosis and inflammation in NAFLD [67]. TSPO ligands exert hepatoprotective effects in experimental models of liver injury, and the TSPO ligand koumine improves survival and reduces liver damage in sepsis-associated hepatic injury [68]. Notably, increased TSPO expression correlates with disease progression from simple steatosis to NASH, while TSPO deficiency elicits compensatory antioxidant responses that may ultimately be overwhelmed by excessive free cholesterol accumulation [69].

Beyond mitochondria, TSPO-dependent handling of cholesterol at endoplasmic reticulum–mitochondria contact sites may alter cellular cholesterol pools and lipid organisation, with potential effects on lipid-sensing pathways such as SCAP-SREBP1-mediated regulation of lipogenesis and cholesterol biosynthesis. Perturbations in this integrated network have clear physiological consequences. In cardiomyocyte-specific PCSK9 knockout mice (CM-Pcsk9–/–), loss of PCSK9 resulted in increased TSPO expression, mitochondrial cholesterol accumulation, enhanced ER-mitochondria contacts, impaired mitochondrial bioenergetics, and reduced cardiac function [70]. Mechanistically, PCSK9 promotes TSPO degradation via a GRP78-dependent ubiquitin-proteasome pathway in cardiomyocytes, maintaining appropriate mitochondrial cholesterol levels; excessive TSPO, by contrast, leads to cholesterol overload, altered membrane properties, elevated ROS, and overwhelmed antioxidant defences. These observations suggest that TSPO may act as a regulator linking mitochondrial and cellular cholesterol trafficking to redox balance(to be discussed in the next paragraph) and cellular stress responses, with important implications for conditions characterised by oxidative stress and mitochondrial dysfunction, including heart failure, metabolic disease, and neurodegeneration.

Together, these findings support a revised model in which TSPO may integrate cholesterol sensing, enzymatic control of porphyrin metabolism, and redox regulation. This integrated role may influence steroidogenic, metabolic, and inflammatory pathways in a tissue- and context-dependent manner, thereby extending its biological relevance beyond classical steroidogenesis; however, this remains to be fully validated.

## TSPO in redox and calcium (Ca^2+^) signalling

TSPO participates in mitochondrial redox and Ca^2+^ signalling through interactions with gatekeeping proteins (see Section TSPO Interactions in Mitochondrial Gatekeeping; Table 1 summarises TSPO ligands that modulate Ca^2+^ signalling).

**Table 1:** TSPO ligands modulating mitochondrial redox homeostasis and ROS production.

Ligand	Target condition	Mechanism of action	Key findings	Reference
Ro5-4864	Neuropathic pain	Enhances autophagy and mitochondrial dynamics	Improved mitochondrial function and reduced ROS accumulation	Hao et al. [149]
ZBD-2	Postpartum depression	Modulates calcium influx and mitochondrial ROS	Reduced oxidative stress and neuroinflammation	Zhai et al. [50]
PK11195	Neuroinflammatory conditions	Reduces ROS production and mitochondrial damage	Improved cognitive performance	Lan et al. [150]
XBD173	Alzheimer’s disease	Enhances mitochondrial biogenesis	Reduced mitochondrial dysfunction and oxidative stress	Lauren H Fairley et al. [85]
Etifoxine	Peripheral neuropathy	Enhances antioxidant defense mechanisms	Reduced ROS levels and improved mitochondrial health	Awad-Igbaria et al. [151]
SSR180575	Ischaemic cardiac conditions	Preserves mitochondrial integrity	Reduced ROS accumulation and improved cardiac resilience	Leducq et al. [152]

TSPO expression is markedly increased during myocardial ischaemia/reperfusion (I/R) injury. In H9c2 cardiomyocytes exposed to anoxia/reoxygenation (A/R), TSPO knockdown reduces oxidative stress by limiting reactive oxygen species (ROS) production, preserving mitochondrial membrane potential (ΔΨm), inhibiting mitochondrial permeability transition pore (mPTP) opening, and ultimately decreasing apoptosis. TSPO-dependent regulation of ROS has also been implicated in neuroinflammatory disorders [50] demonstrated that TSPO interacts with translocase of the outer mitochondrial membrane 40 (TOM40) to modulate mitochondrial Ca^2+^ influx, thereby preserving mitochondrial integrity and reducing oxidative stress. In the same study, the TSPO ligand ZBD-2 suppressed microglial overactivation in the hippocampus and amygdala. This attenuated ROS production and inflammatory responses [50, 71, 72].

Pharmacological modulation of TSPO has therefore been extensively explored in the context of oxidative stress, often using ROS levels as a functional read-out of ligand efficacy (Table 1). The TSPO ligand PK11195 reduces ROS generation and improves mitochondrial membrane potential in models of neuroinflammation and neurodegeneration [73]. Similarly, etifoxine enhances antioxidant defence mechanisms while reducing ROS levels, conferring neuroprotection in experimental models of peripheral neuropathy. However, TSPO upregulation can also exacerbate oxidative stress under specific conditions. In astrocytes, for example, elevated TSPO expression alters glucose metabolism. It promotes pro-inflammatory signalling, leading to increased ROS production and mitochondrial dysfunction, highlighting the context-dependent nature of TSPO function.

The relationship between TSPO and ROS appears to be bidirectional. Increased mitochondrial ROS generation is associated with suppression of PARK2-mediated ubiquitination. This sustains TSPO re-expression by activating the ROS-sensitive kinase PRKCE, thereby establishing a retrograde-like signalling loop that impairs mitochondrial quality control [74]. Consistent with this mechanism, treatment with the mitochondrial uncoupler carbonyl cyanide 4-(trifluoromethoxy)phenylhydrazone (FCCP) induces TSPO accumulation, likely due to elevated ROS following acute mitochondrial uncoupling. As outlined above, TSPO suppresses mitophagy by interfering with PARK2-dependent mitochondrial ubiquitination, leading to further accumulation of ROS.

**Fig. 2:**
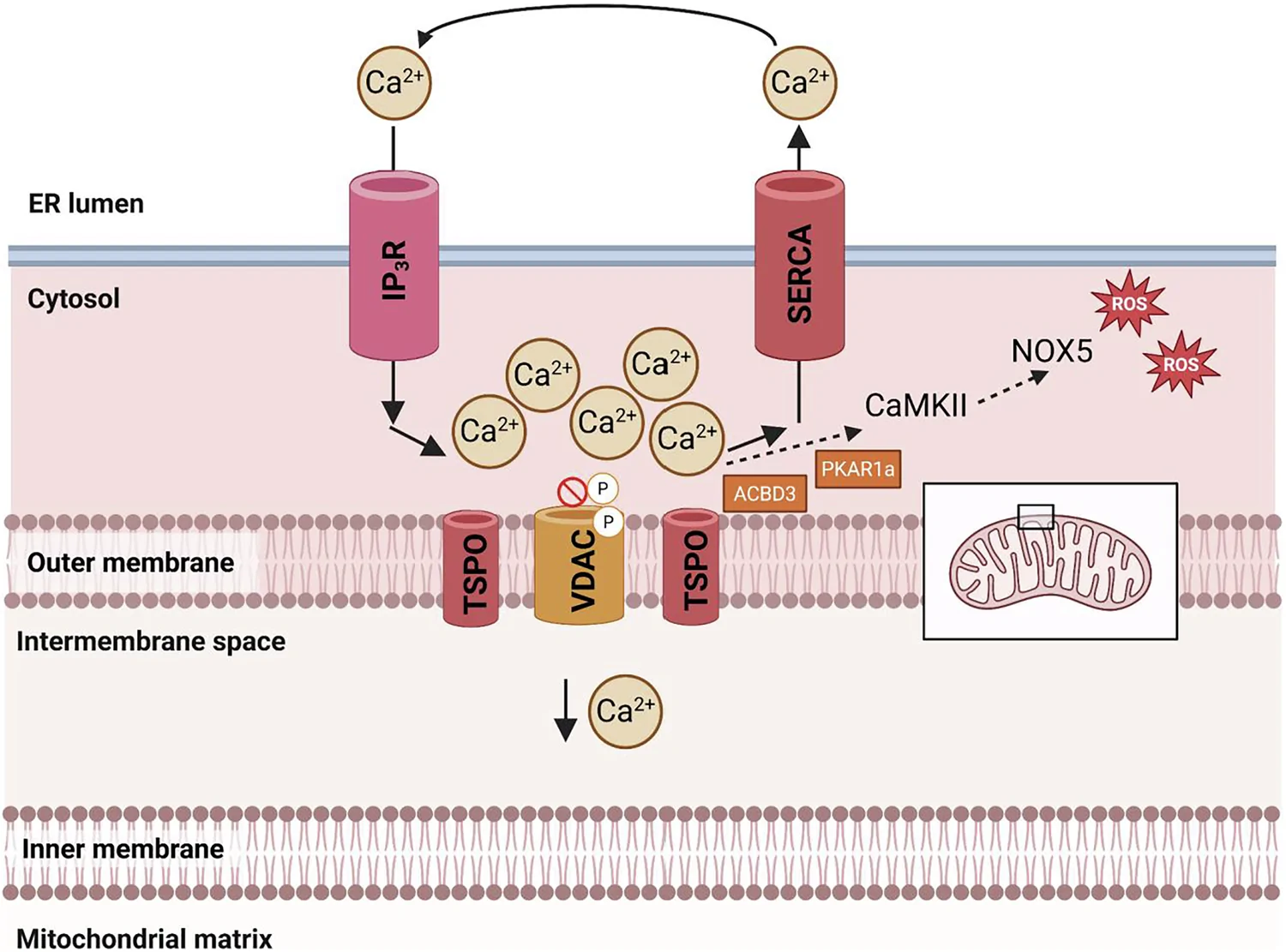
TSPO in redox stress and mitochondrial Ca^2+^ signalling. Mechanistically, TSPO overexpression promotes recruitment of ACBD3 and PKA to the outer mitochondrial membrane. This enhances VDAC1 phosphorylation and reduces its Ca^2+^ permeability, thereby impairing mitochondrial calcium uptake and causing cytosolic Ca^2+^ accumulation. Elevated cytosolic Ca^2+^ subsequently activates CaMKII-NOX signalling, further amplifying ROS production. Through this mechanism, TSPO links mitochondrial Ca^2+^ handling to oxidative stress signalling. However, elevated mitochondrial cholesterol levels may be sufficient to deplete GSH pools, thereby leading to oxidative stress. Created with BioRender.com.

Building on its interactions with mitochondrial gatekeeping proteins (see Section ‘TSPO Interactions in Mitochondrial Gatekeeping’), TSPO regulates Ca^2+^ signalling through modulation of VDAC1 activity. Mechanistically, TSPO promotes recruitment of ACBD3 and PKA to the outer mitochondrial membrane, resulting in phosphorylation of VDAC1 and reduced Ca^2+^ permeability [75]. This attenuation of mitochondrial Ca^2+^ uptake leads to elevated cytosolic Ca^2+^ levels, which activate Ca^2+^-dependent enzymes such as NADPH oxidases (NOX), thereby enhancing reactive oxygen species (ROS) production. Thus, TSPO-mediated regulation of Ca^2+^ signalling is tightly coupled to cellular redox control. A graphical summary of this pathway is provided in Fig. 2.

**Fig. 3:**
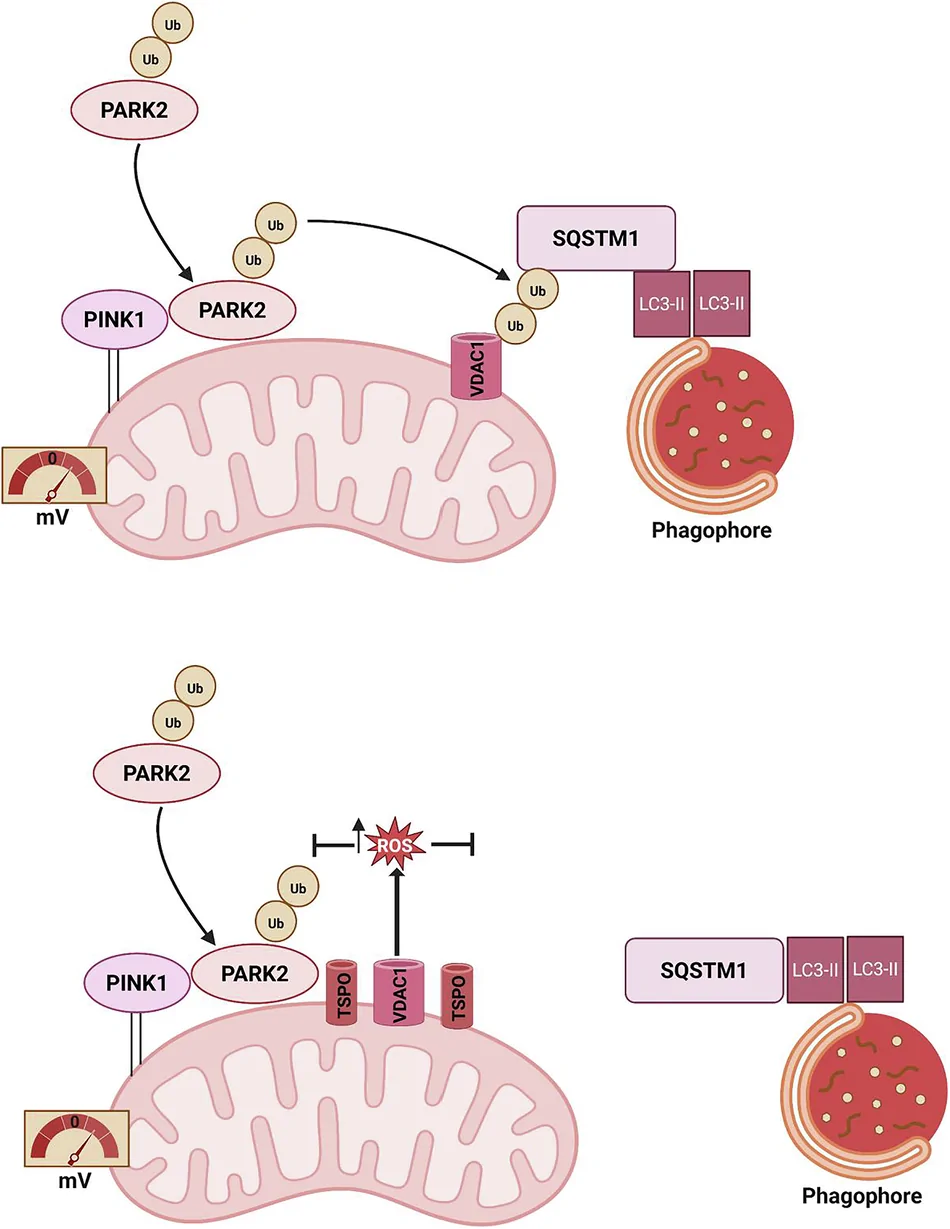
TSPO-dependent regulation of autophagy and mitophagy. TSPO modulates general autophagic processes by influencing mitochondrial reactive oxygen species (ROS) production, mitochondrial membrane permeability, and metabolic stress signalling. Under conditions of low TSPO expression relative to VDAC1, ROS generation is limited, which supports efficient autophagic flux and cellular homeostasis. In the context of mitophagy, low TSPO: VDAC1 ratios enable PARK2 (Parkin)-mediated ubiquitination of outer mitochondrial membrane proteins and the recruitment of the autophagosomal machinery, including SQSTM1/p62, thereby promoting the selective clearance of damaged mitochondria. Conversely, a higher TSPO: VDAC1 ratio increases ROS production, which inhibits PARK2-dependent ubiquitination, impairs SQSTM1/p62 recruitment, and suppresses mitophagy, thereby linking TSPO activity to mitochondrial quality control. This model underscores TSPO as a critical regulator that connects mitochondrial cholesterol, ROS signalling, and autophagic/mitophagic pathways. Created with BioRender.com.

Accordingly, Ca^2+^ homeostasis has emerged as both a functional read-out and a therapeutic target for TSPO ligands (Table 2). Several TSPO ligands modulate mitochondrial Ca^2+^ dynamics while concomitantly reducing ROS production. PK11195 attenuates mitochondrial Ca^2+^ accumulation, preserves mitochondrial function, and protects against neuroinflammation and amyloid-β toxicity in models of Alzheimer’s and Parkinson’s disease [76]. XBD173 alleviates anxiety and post-traumatic stress disorder (PTSD) by enhancing neurosteroidogenesis and regulating Ca^2+^-dependent neurotransmission, thereby improving stress resilience (Fig. 3).

**Table 2:** TSPO ligands regulating mitochondrial and cellular Ca^2+^ signalling.

Ligand	Target condition	Mechanism of action	Key findings	Reference
PK11195	Neurodegenerative Disorders (Alzheimer’s, Parkinson’s)	Reduces mitochondrial Ca2+ overload, stabilizes mitochondrial function	Mitigates neuroinflammation and protects against amyloid-beta toxicity	Ma, L., et al. [153]
XBD173	Anxiety, PTSD	Enhances TSPO-mediated neurosteroidogenesis, increasing GABA-mediated inhibitory neurotransmission	Reduced panic- and anxiety-like behaviour in rodents and showed anxiolytic efficacy in humans without benzodiazepine-like sedation, tolerance, or withdrawal.	Rupprecht et al. [154]
Etifoxine	Stroke	Limits Ca²⁺-induced mitochondrial dysfunction through inhibition of mPTP opening and preservation of mitochondrial respiration	Reduces neuroinflammation and protects against ischemic brain injury	Li et al. [155] Palzur et al. [156]
Ro5-4864	Cancer (Glioblastoma,	Modulates TSPO-associated mitochondrial permeability transition pore (mPTP), influencing Ca²⁺-dependent mitochondrial apoptosis	Regulates mitochondrial membrane potential, cytochrome c release, and caspase activation during chemotherapy-induced apoptosis	Mylroie et al. [157]
FGIN-1-27	Neurodegeneration	Improves mitochondrial function, reduces oxidative stress, and indirectly preserves mitochondrial Ca²⁺ homeostasis	Restores mitochondrial membrane potential and respiration, reduces oxidative metabolites, and attenuates astrocyte activation	Zhang et al. [158]
Siramesine	Neuroinflammation, Neurodegenerative Diseases	Modulates cytosolic Ca²⁺ signalling in microglia via extracellular Ca²⁺ influx and endoplasmic reticulum Ca²⁺ release	Elevates intracellular Ca²⁺ in BV2 microglia; uniquely promotes Ca²⁺ mobilization from both extracellular and ER stores, supporting modulation of microglial activation	Lodholz et al. [160]

Etifoxine stabilises mitochondrial Ca^2+^ homeostasis and limits excitotoxic damage in cerebrovascular disease and epilepsy, providing neuroprotection and reducing seizure severity, although it is associated with mild sedative and gastrointestinal side effects [77]. In oncology, the TSPO ligand Ro5-4864 induces Ca^2+^-dependent apoptosis in cancer cells, sensitising tumours to chemotherapy and inhibiting proliferation, particularly in therapy-resistant breast cancer, albeit with dose-limiting neurological side effects. Siramesine, another TSPO-associated ligand, modulates cytosolic Ca^2+^ levels in microglia by promoting Ca^2+^ influx from extracellular and endoplasmic reticulum sources, thereby shaping neuroinflammatory responses [78].

However, it should be noted that cholesterol is a key regulator of cellular and mitochondrial redox balance, modulating the activity of membrane-associated antioxidant systems, including superoxide dismutases and glutathione peroxidase, by influencing membrane fluidity and lipid raft composition [79]. TSPO-mediated cholesterol redistribution can indirectly influence antioxidant capacity and ROS generation [80]. Dysregulation of TSPO-mediated cholesterol trafficking may alter membrane cholesterol content. This can perturb ROS homeostasis and contribute to oxidative stress at both mitochondrial and cellular levels. This suggests that TSPO may exert a dual role in redox regulation by coordinating cholesterol-dependent antioxidant capacity and mitochondrial calcium signalling.

## TSPO in autophagy and mitophagy

Autophagy is a fundamental homeostatic process responsible for the lysosomal degradation and recycling of damaged proteins and organelles, thereby preserving cellular integrity, metabolic balance, and adaptive stress responses. In the nervous system, efficient autophagic flux is particularly critical, as post-mitotic neurons rely heavily on this pathway to sustain long-term viability, synaptic plasticity, and resilience to metabolic and oxidative stress [81]. A specialised form of autophagy, mitophagy, selectively targets damaged or dysfunctional mitochondria for degradation, ensuring mitochondrial quality control and preventing excessive reactive oxygen species (ROS) production and bioenergetic failure. Disruption at any stage of autophagy or mitophagy, including cargo recognition, autophagosome formation, lysosomal fusion, or degradation, can impair organelle turnover. This contributes to the onset and progression of neurodegenerative and neuroinflammatory diseases.

TSPO has emerged as a context-dependent modulator of autophagy, with evolutionarily conserved yet context-specific roles across biological systems. In plants, particularly *Arabidopsis thaliana*, TSPO contains a functional autophagy-interacting motif (AIM) that enables direct binding to ATG8 proteins, allowing TSPO to act as a selective autophagy receptor. Under stress conditions-especially those involving heme accumulation-plant TSPO promotes the autophagic degradation of specific substrates, thereby protecting cellular homeostasis [82]. These findings provided early evidence that TSPO can directly interface with the core autophagic machinery as summarised in Fig. 3.

In mammalian systems, TSPO regulation of autophagy and mitophagy has been primarily linked to mitochondrial ROS signalling. As discussed in preceding sections, TSPO-mediated ROS accumulation suppresses PARK2-dependent ubiquitination. This prevents recruitment of autophagic adaptors such as SQSTM1 (p62). As a result, mitophagy is impaired and dysfunctional mitochondria accumulate [32]. These findings suggest that TSPO may act as a negative regulator of mitophagy under conditions of oxidative stress.

Consistent with the mechanism, exposure to neurotoxins used to model Parkinson’s disease (PD) induces robust TSPO upregulation, resulting in impaired ubiquitin-dependent mitochondrial clearance and maladaptive prodromic signalling [71]. More recently, studies in microglia and retinal models have further refined this concept. In inflammatory settings, TSPO stabilisation has been reported to inhibit PARK2-mediated mitophagy, sustain mitochondrial dysfunction, and amplify NF-κB-dependent pro-inflammatory gene transcription, thereby perpetuating chronic neuroinflammation and excitotoxicity [72]. These observations highlight a disease-relevant role for TSPO in linking defective mitophagy to innate immune activation in the central nervous system.

Beyond its interaction with the PINK1–PARK2 axis, TSPO also interfaces with metabolic regulators of autophagy. TSPO interacts with the acyl-CoA-binding protein (ACBP/ACBD1), thereby influencing intracellular acetyl-CoA availability. This interaction also affects mTOR signalling [83]. In TSPO-deficient rat models, altered acetyl-CoA levels reduce Raptor acetylation, leading to suppression of mTORC1 activity and disinhibition of ULK1-dependent autophagy initiation. These findings position TSPO as a metabolic rheostat that couples mitochondrial status to nutrient-sensitive autophagic control.

Importantly, elevated TSPO expression does not universally suppress autophagy. In models of cerebral ischaemia-reperfusion injury, TSPO expression is markedly increased and correlates with excessive, dysregulated autophagy, characterised by elevated Beclin-1 and LC3B levels, reduced p62, and impaired autophagic flux. TSPO downregulation in this context prevents autophagy hyperactivation, reduces neuronal injury, and attenuates ROS production and pro-inflammatory cytokine release, including TNF-α and IL-6, identifying TSPO as a promising therapeutic target in ischaemic stroke [84]. These data underscore that TSPO can either restrain or exacerbate autophagy depending on the cellular stress landscape.

Additional insight into TSPO-mediated control of autophagy and mitophagy has been gained through pharmacological studies. The TSPO ligand Ro5-4864 reduces mechanical allodynia in spinal nerve ligation models by enhancing autophagic flux, as evidenced by increased ATG7, Beclin-1, and LC3 levels alongside reduced p62 accumulation. Mechanistically, Ro5-4864 activates the SIRT1/PGC-1α signalling axis, preventing autophagy impairment while simultaneously promoting mitochondrial biogenesis [68]. Similarly, Ro5-4864 and XBD173 restore mitophagy in Alzheimer’s disease models by improving SQSTM1/p62-dependent cargo recognition, preserving PINK1 and PARK2 expression, reducing mitochondrial fragmentation, and normalising LC3 dynamics in amyloid precursor protein (APP)-overexpressing cells [85].

Consistent with these findings, PK11195 confers neuroprotection in lipopolysaccharide-induced cognitive impairment by attenuating microglial activation, restoring autophagic balance, and improving learning and memory performance [73]. Collectively, these pharmacological studies demonstrate that TSPO ligands can recalibrate autophagy and mitophagy in disease-relevant contexts, often coupling mitochondrial quality control to anti-inflammatory and neuroprotective outcomes (please see Table 3).

**Table 3:** TSPO ligands influencing autophagy and mitochondrial quality control.

Ligand	Target condition	Mechanism of action	Key findings	Reference
Ro5-4864	Alzheimer’s disease	Modulates P62/SQSTM1 autophagic cargo receptor	Attenuates mitophagy deficits and mitochondrial fragmentation	Fairley et al. [85]
XBD173	Alzheimer’s disease	Modulates P62/SQSTM1 autophagic cargo receptor	Reduces mitochondrial fragmentation without altering PARK2/PINK1/LC3	Fairley et al. [85]
PK11195	Neuroinflammatory conditions	Suppresses TSPO-mediated microglial activation and excessive autophagy	Reduces mitochondrial damage and improves cognitive function	Lan et al. [150}
Ro5-4864	Neuropathic pain (NP)	Enhances autophagy and SIRT1/PGC-1α-mediated mitochondrial biogenesis	Restores autophagy flux, promotes mitochondrial biogenesis, and alleviates neuropathic pain	Hao et al. [149]
PK11195	Hepatocellular carcinoma	Induces ferroptosis and enhances antitumor immunity	Suppresses tumor growth and potentiates anti-PD-1 immunotherapy	Zhang et al. [159]

Taken together, these findings support the view that TSPO acts as a context-dependent modulator of autophagy and mitophagy, capable of either impairing mitochondrial quality control under chronic inflammatory and oxidative stress or restoring autophagic balance when appropriately targeted. Positioning of TSPO as a critical molecular switch linking mitochondrial dysfunction, immune activation, and neurodegeneration is significant.

## TSPO and inflammation

TSPO is a well-established molecular signature of immune activation and inflammation, with prominent expression in both central and peripheral immune cells, including microglia, macrophages, astrocytes, and lymphocytes. Its upregulation during inflammatory states suggests a close association with immune cell metabolism, mitochondrial function, and redox signalling [86]. As such, TSPO has gained recognition as a biomarker of inflammation and a potential therapeutic target in autoimmune, neuroinflammatory, and metabolic disorders [13].

In immune cells, TSPO has been implicated in regulating key effector functions, including migration, phagocytosis, cytokine secretion, and reactive oxygen species (ROS) production [54, 80]. Within the central nervous system (CNS), TSPO is markedly upregulated in activated microglia during neuroinflammatory processes associated with neurodegenerative diseases, traumatic brain injury, and neuropsychiatric disorders [87]. Activated microglia undergo morphological and metabolic reprogramming, migrating toward sites of neuronal damage and releasing both neurotoxic and neuroprotective mediators [88].

Importantly, TSPO expression appears to be selectively associated with pro-inflammatory microglial phenotypes. Experimental studies demonstrate that TSPO expression is highest in M1-polarised microglia stimulated with lipopolysaccharide (LPS), interleukin-1β (IL-1β), or interferon-γ (IFN-γ), while anti-inflammatory M2 microglia show minimal TSPO induction [87]. TSPO modulation has been reported to influence cytokine secretion profiles via mitochondrial ROS signalling and NF-κB pathway activation, underscoring its role as a regulator of inflammatory signalling at the mitochondrial level [86]. Conversely, TSPO knockdown exacerbates inflammatory responses, further supporting its functional involvement in immune regulation [89].

Beyond the CNS, TSPO also contributes to systemic immune alterations. In Parkinson’s disease and other neurodegenerative disorders, changes in peripheral immune cell populations, including altered T-lymphocyte counts and CD4:CD8 ratios, suggest that TSPO-mediated immune modulation extends beyond the brain [90]. However, species-specific differences and off-target effects of TSPO ligands complicate interpretation, as highlighted by studies showing TSPO-independent pharmacological actions of classical ligands such as PK11195.

TSPO’s involvement in inflammation is further intertwined with cholesterol metabolism and metabolic homeostasis. Dysregulated TSPO expression and cholesterol transport have been implicated in obesity, insulin resistance, and type 2 diabetes, with reduced TSPO expression observed in adipose tissue and skeletal muscle in metabolic disease states [91]. These findings suggest that TSPO links mitochondrial metabolism, lipid handling, and inflammatory signalling across immune and metabolic tissues.

Therapeutically, TSPO ligands exhibit anti-inflammatory and antioxidant properties, reducing microglial activation, oxidative stress, and cytokine production in preclinical models [92]. TSPO modulation has shown protective effects in models of neurodegeneration, inflammatory pain, and autoimmune disease, reinforcing its translational potential. Nevertheless, the development of selective TSPO ligands with favourable pharmacokinetics and minimal off-target activity remains a critical challenge. Well-designed clinical trials are urgently needed to determine the therapeutic efficacy and safety of TSPO-targeted interventions in inflammatory and immune-mediated diseases [93].

Overall, these insights position TSPO not only as a biomarker of immune activation but also as a dynamic regulator of inflammation that integrates mitochondrial metabolism with cell signalling, representing a promising yet complex target for novel anti-inflammatory therapies.

## TSPO and neurological disorders

Research on the translocator protein (TSPO) in the central nervous system (CNS) has largely focused on its association with neuroinflammation and neurodegeneration, particularly microglial activation. Microglia, the resident macrophages of the CNS, constitute the first line of immune defence and play a central role in inflammatory responses to injury and disease [94]. TSPO is highly expressed in activated microglia and has been extensively implicated in processes such as oxidative stress, mitochondrial dysfunction, and neurodegeneration, positioning it as both a biomarker and a potential therapeutic target across a range of neurological disorders [95].

Given its robust upregulation in activated microglia, TSPO has been widely exploited as a molecular target for positron emission tomography (PET) imaging to monitor neuroinflammation in vivo. First- and second-generation TSPO ligands have enabled visualisation of inflammatory responses in neurodegenerative and oncological conditions, including Alzheimer’s disease (AD), Parkinson’s disease (PD), gliomas, and multiple sclerosis (MS) [96]. Preclinical studies using rat glioma models demonstrated significant uptake of TSPO ligands, such as ^18F-PBR06 and ^18F-DPA714, within tumours compared with contralateral brain regions, confirming high TSPO specificity [97]. However, a key limitation of TSPO imaging is its inability to distinguish TSPO expression in tumour cells from that in infiltrating inflammatory cells.

Clinical translation of TSPO imaging has been complicated by the presence of a common TSPO polymorphism (A147T), which significantly reduces the binding affinity of several classical ligands, including [^11C]PK11195 [98]. This polymorphism is present in ~30% of individuals of European ancestry [99], influences ligand binding whilst affecting diurnal cortisol rhythms [100]. To address the limitation, novel TSPO ligands with polymorphism-independent binding profiles have been developed, improving the reliability of TSPO PET imaging across diverse populations [101].

Beyond biomarking via imaging, TSPO, by affecting mitochondrial homeostasis, redox balance, and mitophagy, is core to neurodegenerative diseases such as AD and PD [71, 102] and its pharmacological modulation demonstrated neuroprotective effects in multiple preclinical models. The TSPO ligand PK11195 ameliorated behavioural deficits and reduced β-amyloid pathology in 3xTg-AD rats [103], while XBD173 improved clinical symptoms in patients with multiple sclerosis [104]. Similarly, Ro5-4864 conferred protection against β-amyloid toxicity in hippocampal cultures [105]. Many of these effects are thought to be mediated by enhanced neurosteroidogenesis, with neurosteroids acting as inhibitory neuromodulators and contributing to anxiolytic and neuroprotective outcomes [106, 107].

Although TSPO expression in mature neurons under physiological conditions remains low and difficult to detect, growing evidence supports a role for TSPO in neurogenesis and neuronal differentiation. TSPO has been identified in neural stem cells (NSCs), particularly within neurogenic niches, and its endogenous ligand, diazepam-binding inhibitor (DBI), has been shown to promote stem cell proliferation and expansion [108]. While DBI can also interact with GABA_A receptors, suggesting indirect mechanisms, these findings raise the possibility that TSPO participates in regulating early neural development and differentiation through both mitochondrial and neurotransmitter-related pathways [109].

Consistent with the notion, several in vitro studies have demonstrated that TSPO ligands influence neuronal differentiation. PK11195 induced neurite outgrowth and increased β-III tubulin expression in PC12 cells without additional differentiation cues, while similar morphological changes were observed in N1E-115 neuroblastoma cells [110]. In P19 embryonic carcinoma cells, PK11195 increased the expression of multiple neuronal markers, albeit less robustly than retinoic acid [111]. Collectively, these findings suggest that TSPO modulation may bias cell fate decisions toward neuronal lineages.

TSPO has also been implicated in neural repair and regeneration, particularly within the peripheral nervous system (PNS). Ro5-4864 and etifoxine enhanced nerve regeneration and remyelination following peripheral nerve injury, with reported increases in neurite outgrowth and functional recovery [112]. While some studies observed concomitant increases in brain-derived neurotrophic factor (BDNF) in models of traumatic injury, TSPO expression was upregulated in damaged neurons, further supporting a context-dependent role for TSPO in neuronal stress and repair [113].

In AD, TSPO PET imaging consistently reveals elevated tracer binding in regions associated with amyloid and tau pathology, reflecting microglial activation and neuroinflammation [114, 115]. However, whether TSPO expression spatially correlates with amyloid or tau deposition remains controversial, and some clinical studies have reported limited diagnostic discrimination between AD patients and age-matched controls [116]. Beyond inflammation, TSPO has been linked to amyloid-induced mitochondrial dysfunction and neuronal death [105, 117].

In PD, TSPO expression is increased in the substantia nigra and basal ganglia, correlating with microglial activation and disease severity, particularly at early stages [118]. PET imaging studies have revealed region-specific increases in tracer uptake, although results vary depending on disease stage and radioligand used [119]. These findings support the use of TSPO PET as a tool for monitoring neuroinflammatory dynamics during PD progression.

TSPO is similarly upregulated in active lesions of multiple sclerosis, where it serves as a marker of inflammation and demyelination [120]. Preclinical and clinical PET studies with TSPO radiotracers have demonstrated reduced tracer uptake after immunosuppressive therapy, highlighting TSPO PET as a promising noninvasive biomarker of treatment efficacy and disease activity [121].

Finally, TSPO expression is elevated following acute CNS injuries such as stroke and traumatic brain injury (TBI), particularly in peri-infarct regions, where it reflects microglial activation and secondary inflammatory responses [122]. TSPO ligands have shown neuroprotective effects in these conditions, reducing lesion size, attenuating inflammation, and improving functional recovery [5, 91].

Collectively, these observations highlight TSPO as a multifaceted regulator in neurological disorders, integrating neuroinflammatory, mitochondrial, and neuroprotective pathways, and underscore its potential as both a diagnostic biomarker and a therapeutic target across CNS diseases.

## TSPO and psychiatric illnesses

Accumulating evidence indicates that dysregulated TSPO expression and function associate with a wide range of psychiatric disorders, including anxiety, depression, schizophrenia, bipolar disorder, and substance use disorders [123]. Like neurodegenerative conditions, psychiatric illnesses are increasingly recognised to involve chronic neuroinflammation, microglial activation, mitochondrial dysfunction, and altered lipid and steroid metabolism processes in which TSPO plays a central regulatory role. These shared pathological features support the conceptual continuity between neurological and psychiatric disorders and underscore TSPO’s relevance across the neuropsychiatric disease spectrum.

Genetic variability in TSPO further highlights its contribution to psychiatric phenotypes. In individuals with alcohol use disorder (AUD), the TSPO rs6971 polymorphism has been associated with altered lipid profiles, including total cholesterol, LDL, HDL, and triglycerides. It influences the severity of alcohol withdrawal symptoms, with LDL acting as a mediating factor [124]. These findings parallel observations in neurological disorders, where TSPO polymorphisms affect ligand binding and mitochondrial cholesterol handling, reinforcing the link between TSPO, lipid metabolism, and brain dysfunction.

As in neurodegenerative diseases, TSPO has emerged as both a biomarker of neuroinflammation and a therapeutic target in psychiatric disorders. Preclinical studies demonstrate that TSPO ligands modulate microglial activation, attenuate neuroinflammatory signalling, and improve behavioural outcomes relevant to psychiatric disease models [7, 125]. Beyond immune modulation, TSPO influences mitochondrial function, neurosteroidogenesis, and neurotransmitter systems, providing a mechanistic framework for its pleiotropic effects on mood, anxiety, and cognition [126].

## Anxiety disorders

Anxiety disorders are characterised by persistent fear, hyperarousal, and dysregulated stress responses [127]. Consistent with findings in neurological injury and neurodegeneration, TSPO has been implicated in anxiety through its regulation of mitochondrial function and neurosteroid synthesis, particularly neurosteroids that positively modulate GABA_A receptor signalling. Numerous preclinical studies have demonstrated anxiolytic effects of TSPO ligands across diverse animal models [128]. Multiple structurally distinct classes of TSPO ligands, including isoquinoline carboxamides (e.g., PK11195), benzothiazepines, benzoxazepines, indole acetamides, and pyrazolopyrimidineacetamides, have been shown to reduce anxiety-like behaviours [129]. Notably, the TSPO ligand ZBD-2 attenuated anxiety-like behaviour in a chronic inflammatory pain model without affecting nociceptive thresholds or peripheral inflammation, highlighting a central TSPO-mediated mechanism [130]. Altered TSPO expression in anxiety-related brain regions further supports its involvement in anxiety pathophysiology [131].

## Depression

Major depressive disorder is a complex condition involving dysregulation of neurotransmitter systems, impaired neuroplasticity, mitochondrial dysfunction, and sustained neuroinflammation [132]. In line with observations in Alzheimer’s disease and Parkinson’s disease, PET imaging studies have revealed altered TSPO expression in brain regions associated with mood regulation, consistent with microglial activation in depressed patients [133]. Several TSPO ligands-including FGIN-1-27, XBD173, and PIGA-have demonstrated antidepressant-like effects in preclinical models, likely through suppression of neuroinflammatory signalling and enhancement of neurogenesis and mitochondrial resilience [134]. Recent studies employing global and neuronal TSPO KO mice have further delineated the contribution of neuronal TSPO to anxiety- and depression-related behaviours, underscoring cell type–specific roles analogous to those proposed in neurodegenerative disorders [131]. Additional ligands, such as YLIPA08, have also shown combined anxiolytic and antidepressant efficacy in rodent models of postpartum depression [135]. At present, etifoxine remains the only TSPO ligand approved for clinical use. Clinical studies have demonstrated anxiolytic efficacy comparable to benzodiazepines, such as lorazepam, while avoiding sedative effects, supporting TSPO modulation as a viable therapeutic strategy in affective disorders [136].

## Schizophrenia

Schizophrenia is a severe psychiatric disorder characterised by disturbances in perception, cognition, and emotional regulation [137]. Similar to neurodegenerative diseases, schizophrenia has been associated with neuroinflammatory changes, microglial activation, and altered glutamatergic signalling. TSPO has been implicated through its influence on mitochondrial function, neurosteroidogenesis, and modulation of glutamatergic and GABAergic neurotransmission, including NMDA receptor-related pathways. Altered TSPO expression and ligand binding have been reported in individuals with schizophrenia [138]. In preclinical models, TSPO ligands improved cognitive deficits and normalised neurotransmitter imbalances. Notably, the TSPO ligand XBD173 enhanced GABAergic transmission via neurosteroid synthesis, significantly reducing panic-like responses without sedation, highlighting mechanistic overlap with anxiety and mood disorders [139].

## Bipolar disorder

Recurrent manic and depressive episodes characterise bipolar disorder and are increasingly recognised as a disorder involving immune dysregulation, neuroinflammation, and mitochondrial dysfunction [140]. In parallel with findings in multiple sclerosis and neurodegenerative diseases, altered TSPO expression has been reported in individuals with bipolar disorder. TSPO ligands have demonstrated anti-inflammatory and mood-stabilising effects in preclinical models, reinforcing the therapeutic relevance of targeting microglial activation and inflammatory cytokine production [141]. Specifically, the TSPO ligands 2-Cl-MGV-1 and MGV-1 reduced levels of pro-inflammatory cytokines (IL-1β, IL-6, TNF-α, IFNγ) in activated microglia, consistent with mechanisms proposed for TSPO modulation in neurological disorders [142]. Increased TSPO ligand binding in astrocytes further supports the diagnostic potential of TSPO in neuroinflammatory psychiatric conditions.

Together, these findings position TSPO as a unifying molecular node linking psychiatric and neurological disorders through shared mechanisms involving neuroinflammation, microglial activation, mitochondrial dysfunction, and altered lipid and steroid metabolism. As with neurological diseases, major challenges remain in developing TSPO ligands with optimal selectivity, favourable pharmacokinetic properties, and minimal off-target effects. Addressing these challenges will be essential for translating TSPO-based strategies into clinically effective diagnostics and therapeutics. Further elucidation of TSPO’s cell type-specific functions and molecular interactions will be critical for refining its role in psychiatric disease pathophysiology and for advancing next-generation TSPO-targeted interventions [115].

Ultimately, these data underscore TSPO as an integrator of neuroimmune, mitochondrial, and neurosteroid pathways in psychiatric disorders, highlighting its potential as both a biomarker and a target for innovative interventions.

## TSPO in cancer biology and therapeutic targeting

The upregulation of TSPO in tumour cells has emerged as a prominent hallmark of neoplastic transformation and progression, positioning this mitochondrial protein as both a potential biomarker and a therapeutic target in oncology. Tumorigenesis is accompanied by profound dysregulation of mitochondrial function, cholesterol trafficking, redox homeostasis, and apoptotic signalling processes in which TSPO plays a central regulatory role. Increased TSPO ligand binding capacity and/or elevated TSPO protein levels have been reported across a wide spectrum of malignancies, including gliomas, astrocytomas, colorectal, hepatic, breast, oral cavity, prostate, lung, kidney, and ovarian cancers, underscoring its broad relevance in cancer biology [80].

Altered TSPO expression influences tumour growth, angiogenesis, metabolic adaptation, and resistance to apoptosis. TSPO overexpression has been documented in breast, prostate, kidney, lung, and brain cancers. Reduced expression has been observed in select lung and colon carcinomas, suggesting tumour-type-specific roles and regulatory mechanisms. Importantly, elevated TSPO levels have been associated with aggressive tumour phenotypes, chemotherapy resistance, and poor clinical prognosis in several cancers [30, 143].

Recent advances have expanded the functional landscape of TSPO beyond classical apoptosis regulation, implicating it in mitochondrial retrograde signalling (MRR) and the organisation of mito-nuclear contact sites. These processes enable bidirectional communication between mitochondria and the nucleus, facilitating transcriptional reprogramming in response to metabolic or oxidative stress [144]. Given that MRR supports mitochondrial adaptation and survival under oncogenic stress, TSPO-dependent retrograde signalling may represent a mechanistic link between mitochondrial homeostasis and tumour aggressiveness.

At the cellular level, TSPO has been associated with cancer cells by regulating mitochondrial bioenergetics, oxidative stress responses, and membrane permeability [75]. TSPO overexpression has been reported to suppress intrinsic apoptosis by modulating mitochondrial membrane permeability and inhibiting caspase activation, thereby promoting resistance to cytotoxic therapies [145]. These properties reinforce TSPO’s relevance as a prognostic marker and therapeutic vulnerability.

From a translational perspective, TSPO ligands have demonstrated antiproliferative and pro-apoptotic effects across multiple cancer models. Notably, lorazepam, an established benzodiazepine with moderate TSPO affinity, reduces prostate cancer cell proliferation in vitro and tumour growth in xenograft models, concomitant with a reduction in Ki-67 expression [146]. In breast cancer cells, TSPO ligands exhibit biphasic effects, stimulating proliferation at nanomolar concentrations and exerting antiproliferative effects at micromolar concentrations. Novel TSPO-targeted compounds, including carborane-containing ligands, have shown high-affinity binding and efficient cellular uptake in glioma, breast cancer, and colon adenocarcinoma models, highlighting their therapeutic promise [147].

Beyond therapy, TSPO PET imaging has emerged as a non-invasive strategy for tumour detection, staging, and patient stratification based on TSPO expression status [148]. Moreover, TSPO ligands may function as adjuvants to conventional chemotherapy or immunotherapy. Recent evidence suggests that TSPO ligands can modulate immune cell activity, including the suppression of B-cell activation and antibody production, raising the possibility of synergistic integration with immune checkpoint inhibitors. Collectively, these findings position TSPO as a multifunctional regulator of tumour biology and a compelling target for next-generation anticancer strategies, pending rigorous clinical validation.

Future studies integrating TSPO-targeted imaging, selective ligands, and combination therapies will be critical to fully exploit its diagnostic, prognostic, and therapeutic potential in precision oncology.

## Conclusions and future perspectives

TSPO has emerged as an important component of mitochondrial and cellular physiology, although its precise functions remain incompletely defined. Positioned on the outer mitochondrial membrane, TSPO has been implicated in processes such as autophagy, mitophagy, redox homeostasis, calcium signalling, steroid metabolism, and apoptosis. Together, these roles underscore the importance of TSPO in maintaining cellular integrity. Dysregulation of TSPO is implicated in a broad spectrum of pathologies, ranging from neurodegenerative, psychiatric, and metabolic disorders to cancer and inflammatory diseases, underscoring its potential as both a diagnostic biomarker and a therapeutic target.

Recent studies reveal that TSPO’s effects are highly context-dependent. It can promote or inhibit autophagic flux depending on cellular stress, disease state, and tissue type, linking it to disease progression and cellular resilience. Its modulation of reactive oxygen species (ROS), mitochondrial membrane permeability, and interactions with channels such as VDAC1 and TOM40 are critical for redox balance, mitochondrial quality control, and cell survival. TSPO ligands-including PK11195, XBD173, and Ro5-4864-demonstrate neuroprotective, anti-inflammatory, and anticancer properties in preclinical models, while TSPO’s regulation of mitochondrial calcium dynamics connects it to neuronal excitability, synaptic function, and cell fate decisions, highlighting its broad relevance across neurological, psychiatric, and oncological disorders.

Despite these advances, several critical knowledge gaps remain. The precise molecular mechanisms underlying TSPO’s pleiotropic functions, including ligand-specific effects, structural dynamics, and cell-type-specific signalling, remain incompletely elucidated. Its dualistic effects-protective in some contexts, deleterious in others-necessitate precision approaches that account for tissue specificity, disease stage, and microenvironmental cues. Translational challenges also include the development of highly selective TSPO ligands with favourable pharmacokinetics, overcoming polymorphism-related variability in ligand binding, and establishing reliable biomarkers for TSPO activity in humans.

Looking ahead, integrative approaches that combine structural biology, multi-omics profiling, advanced imaging, and functional genomics will be essential. These approaches will help map TSPO interaction networks and regulatory circuits. Such efforts promise to clarify how TSPO coordinates mitochondrial quality control, neuroinflammatory signalling, metabolic adaptation, and cellular stress responses. Clinically, rationally designed TSPO-targeted therapies, potentially in combination with immunomodulators, neurosteroids, or metabolic interventions, offer the prospect of precision medicine strategies for complex diseases. Ultimately, TSPO represents a versatile molecular hub at the crossroads of mitochondrial function, cellular homeostasis, and disease pathogenesis, and continued mechanistic and translational research may transform it from a mechanistic biomarker into a therapeutic linchpin, enabling innovative treatments across neurodegenerative, psychiatric, metabolic, and oncological disorders.
